# Comprehensive review for aflatoxin detoxification with special attention to cold plasma treatment

**DOI:** 10.1007/s12550-025-00582-5

**Published:** 2025-02-01

**Authors:** Yehia A.-G. Mahmoud, Nehal E. Elkaliny, Omar A. Darwish, Yara Ashraf, Rumaisa Ali Ebrahim, Shankar Prasad Das, Galal Yahya

**Affiliations:** 1 Botany and Microbiology Department, Faculty of Science, Tanta University, Tanta, 31527 Egypt; 2 Botany and Microbiology Department, Faculty of Science, Assiut University, Assiut, 71515 Egypt; 3 Applied and Analytical Microbiology Department, Faculty of Science, Ain Shams University, Ain Shams, 11772 Egypt; 4 Cell Biology & Molecular Genetics, Yenepoya Research Centre, Yenepoya (Deemed to Be University), Mangalore, 575018 Karnataka India; 5 Department of Microbiology and Immunology, Faculty of Pharmacy, Zagazig University, Zagazig, Al Sharqia 44519 Egypt; 6 Molecular Biology Institute of Barcelona (IBMB), CSIC, Barcelona, Spain

**Keywords:** Aflatoxins, Carcinogens, Food safety, Cold plasma, Public health, Detoxification

## Abstract

Aflatoxins are potent carcinogens and pose significant risks to food safety and public health worldwide. Aflatoxins include Aflatoxin B1 (AFB1), Aflatoxin B2 (AFB2), Aflatoxin G1 (AFG1), Aflatoxin G2 (AFG2), and Aflatoxin M1 (AFM1). AFB1 is particularly notorious for its carcinogenicity, classified as a Group 1 human carcinogen by the International Agency for Research on Cancer (IARC). Chronic exposure to aflatoxins through contaminated food and feed can lead to liver cancer, immunosuppression, growth impairment, and other systemic health issues. Efforts to mitigate aflatoxin contamination have traditionally relied on chemical treatments, physical separation methods, and biological degradation. However, these approaches often pose challenges related to safety, efficacy, and impact on food quality. Recently, cold plasma treatment has emerged as a promising alternative. Cold plasma generates reactive oxygen species, which effectively degrade aflatoxins on food surfaces without compromising nutritional integrity or safety. This review consolidates current research and advancements in aflatoxin detoxification, highlighting the potential of cold plasma technology to revolutionize food safety practices. By exploring the mechanisms of aflatoxin toxicity, evaluating existing detoxification methods, and discussing the principles and applications of cold plasma treatment.

**Graphical Abstract:**

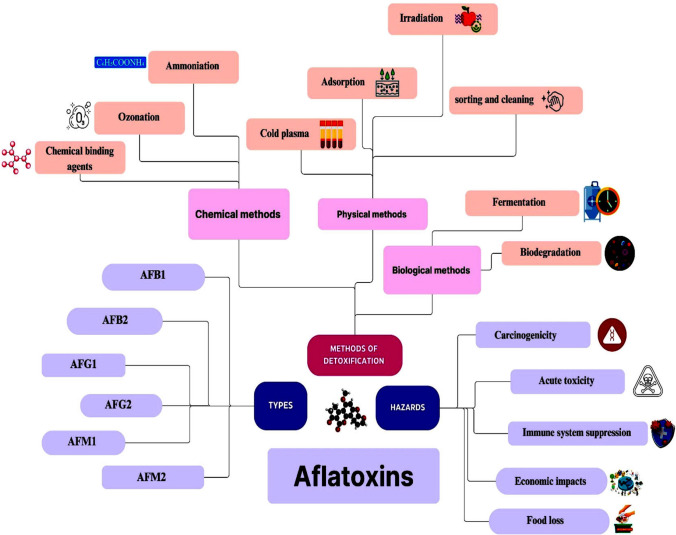

**Supplementary Information:**

The online version contains supplementary material available at 10.1007/s12550-025-00582-5.

## Introduction

Aflatoxins are highly toxic and carcinogenic secondary metabolites produced primarily by certain species of fungi belonging to the *Aspergillus* genus. These moulds commonly infect a wide range of agricultural commodities such as corn, peanuts, cottonseed, and tree nuts, particularly in warm and humid environments. The contamination of food and feed with aflatoxins poses significant risks to human and animal health globally, making them a critical concern for food safety and regulatory authorities. The most studied and problematic aflatoxins include Aflatoxin B1 (AFB1), Aflatoxin B2 (AFB2), Aflatoxin G1 (AFG1), Aflatoxin G2 (AFG2), Aflatoxin M1 (AFM1) and Aflatoxin M2 (AFM2). Among these, AFB1, which is considered the most potent carcinogen, is also classified as a Group 1 human carcinogen by the International Agency for Research on Cancer (IARC) (Cao et al. 2022; EFSA Panel on Contaminants in the Food Chain (CONTAM) et al. 2020; Sun et al. 2017). Chronic exposure to low levels of aflatoxins through contaminated food and feed can lead to severe health consequences, notably liver cancer, immunosuppression, growth impairment in children, and various other systemic disorders (Benkerroum 2020). Acute aflatoxicosis, resulting from high-dose exposure, can cause acute liver damage, hemorrhaging, and potentially fatal outcomes (Abdel-Daim et al. 2021).

Efforts to mitigate aflatoxin contamination have focused on various detoxification strategies. Chemical methods include the use of chemical agents such as acids, bases, oxidants, and reducing agents to degrade or bind aflatoxins. However, these methods may alter food properties, raise safety concerns, or result in undesirable residues. Also, chemical methods degrade toxins on the surface of contaminated food so, the destruction inside is still a slow process (Kutasi et al. 2021). Physical separation techniques like sorting, washing, milling, microwave heating, irradiation, and pulsed light aim to physically remove contaminated particles or reduce aflatoxin levels. While effective to some extent, these methods may not eliminate aflatoxins and can be labour-intensive (Peng et al. 2023). Biological degradation through utilizing microorganisms or enzymes capable of degrading aflatoxins into less toxic or non-toxic metabolites. This approach shows promise but requires careful optimization and control to ensure effectiveness (Nahle et al. 2022). Despite these efforts, challenges remain in achieving consistently high levels of aflatoxin detoxification while maintaining food quality and safety standards.

Recently, cold plasma treatment has emerged as a promising alternative for aflatoxin detoxification. Cold plasma, also known as non-thermal plasma, is a state of matter generated by applying an electric field to a gas, producing a cocktail of reactive oxygen and nitrogen species, UV radiation, and electric fields. These reactive species have potent antimicrobial and detoxifying properties, capable of degrading aflatoxins on food surfaces without significantly altering the nutritional or sensory qualities of the treated products (Wu et al. 2021). Cold plasma treatment offers several advantages over traditional methods. It can rapidly reduce aflatoxin levels on various food surfaces. Unlike chemical treatments, cold plasma does not leave harmful residues or alter food flavour and texture. Also, it can be applied to a wide range of food products, including grains, nuts, spices, and fruits, offering a versatile solution for aflatoxin control in diverse agricultural settings (Mir et al. 2020).

This review aims to comprehensively explore the hazards associated with aflatoxin contamination, discuss existing methods of detoxification, and critically evaluate the potential of cold plasma treatment as an innovative and effective strategy to mitigate aflatoxin risks in food and feed.

## Aflatoxins overview

Aflatoxins are characterized by a common coumarin-like structure with a fused 2-furanone ring system. The basic structure consists of a lactone ring fused to a dihydrofuran ring, typically with a terminal furan ring. This core structure is responsible for the biological activity and toxicity of aflatoxins (Janik et al. 2020). Oxidative transformations can lead to the production of related metabolites and derivatives, such as Aflatoxin M1 (AFM1) and Aflatoxin Q1 (AFQ1), found as metabolites in animal tissues or derived from environmental modifications (De Oliveira et al. 2023).

### Types of aflatoxins

Aflatoxins are a family of structurally related toxic compounds produced by certain species of *Aspergillus* fungi. The most significant aflatoxins in terms of toxicity and prevalence are Aflatoxin B1 (AFB1), B2 (AFB2), G1 (AFG1), G2 (AFG2), and M1 (AFM1) as shown in Table 1. (Asao et al. 1965; Hafez et al. 2021; Levin 2012; Sun et al. 2017; Tian et al. 2020; Xu et al. 2022a; Zhongzhi & Limiao 2020).

**Table 1:** Main types of aflatoxins

Type	Aflatoxin B1 (AFB1)	Aflatoxin B2 (AFB2)	Aflatoxin G1 (AFG1)	Aflatoxin G2 (AFG2)	Aflatoxin M1 (AFM1)
Chemical structure	AFB1 is a difuranocoumarin, containing a double bond in its furan ring which is responsible for its high reactivity and toxicity. In another words, it composed of cyclopentenone ring fusion to the coumarin Lactone ring	AFB2 is structurally similar to AFB1 but lacks the double bond in the furan ring, making it less reactive	AFG1 has a lactone ring and an additional methoxy group compared to AFB1	AFG2 is similar to AFG1 but lacks the double bond in the furan ring	AFM1 is a hydroxylated metabolite of AFB1
Fluorescence	It fluoresces blue under ultraviolet light, hence the “B” designation	It also fluoresces blue under UV light	Distinct green fluorescence under UV light, hence the “G” designation	It fluoresces green under UV light	–
Toxicity	The most potent aflatoxin and one of the most powerful naturally occurring carcinogens It primarily affects the liver, leading to hepatocellular carcinoma (HCC) in chronic exposure	AFB2 is less toxic than AFB1 but still poses significant health risks, including liver damage and carcinogenicity	AFG1 is also highly carcinogenic, though slightly less potent than AFB1	AFG2 is less toxic compared to AFG1 but still poses health risks. It contributes to the overall toxicity of aflatoxin-contaminated foods	AFM1 is less potent than AFB1 but still considered carcinogenic
Occurrence	Found in a wide range of agricultural products, including corn, peanuts, cottonseed, and tree nuts	Typically found alongside AFB1 in contaminated crops and food products	Commonly found in the same foods as AFB1, particularly in nuts and grains	Found with AFG1 in various agricultural products, especially those stored in warm and humid conditions	Found in milk and dairy products from animals fed with contaminated feed. Also detected in human breast milk if the mother has consumed contaminated food
Detection	• Thin-Layer Chromatography (TLC) is an early method for aflatoxin detection • High-Performance Liquid Chromatography (HPLC) offers high sensitivity and specificity • Enzyme-Linked Immunosorbent Assay (ELISA) is widely used for its simplicity and rapid results • Polymerase Chain Reaction (PCR) is used for detecting aflatoxin-producing molds at the DNA level • ultra performance liquid chromatography tandem mass spectrometry (UPLC–MS/MS) method used for the simultaneous determination of aflatoxins				
Control	• crop rotation, proper irrigation, and timely harvesting to reduce mold growth • Proper drying and storage of crops to maintain low moisture levels • Using non-toxigenic strains of Aspergillus to outcompete toxigenic strains • use of fungicides, although this must be carefully managed to avoid resistance and residue issues				

While the aforementioned aflatoxins are the most significant, there are other minor aflatoxins such as, aflatoxin M2 (AFM2) which is another metabolite found in milk, similar to AFM1 but even less toxic. Aflatoxin B2a and G2a are hydroxylated derivatives of AFB2 and AFG2, respectively, found in the urine of animals and humans exposed to aflatoxins (Carvajal-Moreno 2015).

### Synthesis of aflatoxins

**Fig. 1:**
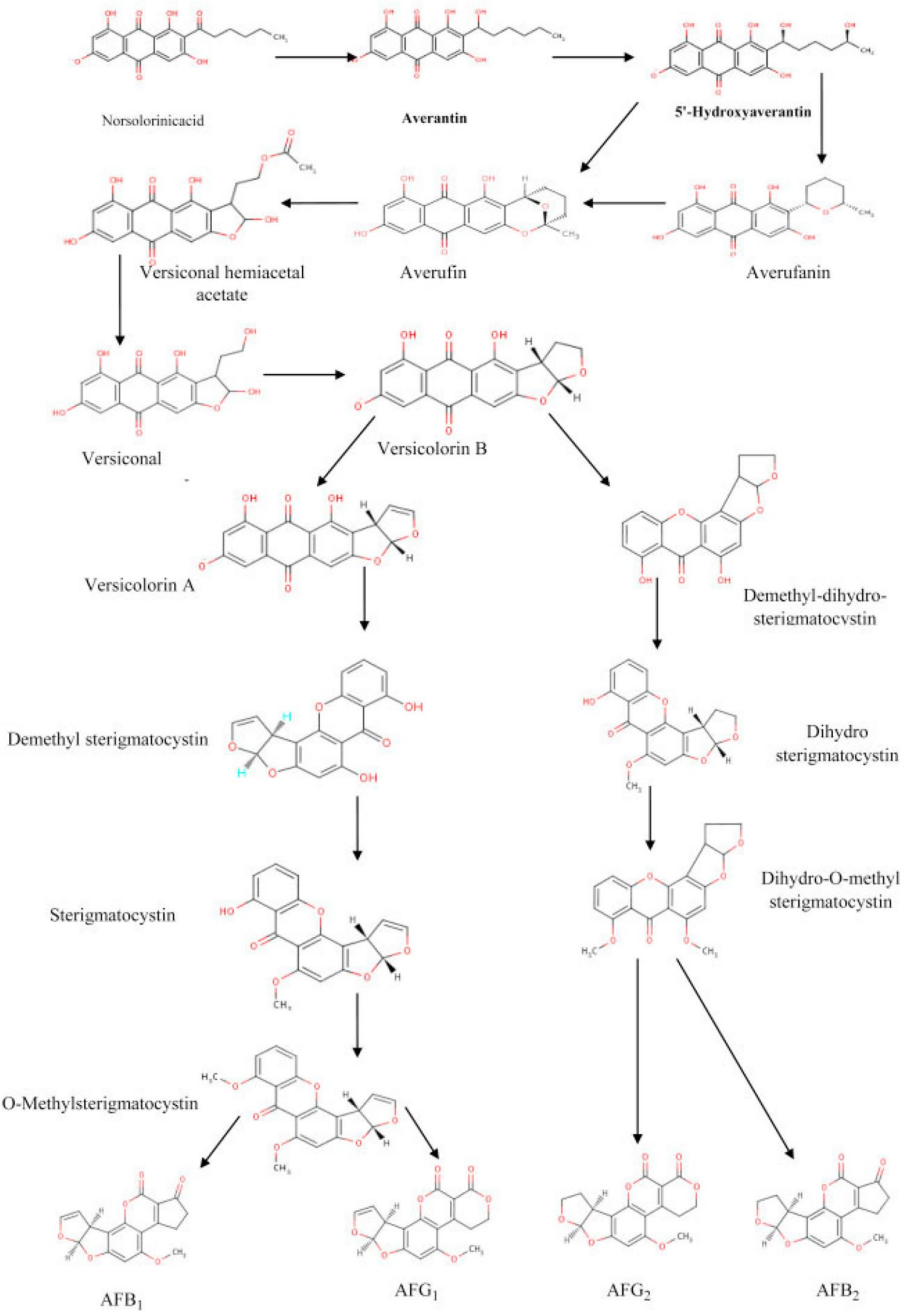
Biosynthesis of aflatoxins

The synthesis of aflatoxins is a complex, multi-step biochemical process carried out by certain species of *Aspergillus* fungi, primarily *Aspergillus flavus* and *Aspergillus parasiticus.* This process involves numerous enzymatic reactions and intermediates, leading to the production of various aflatoxins, including AFB1, AFB2, AFG1, and AFG2. The synthesis begins with the formation of a polyketide chain through the action of polyketide synthase (PKS) enzymes. Acetyl-CoA and malonyl-CoA are the primary building blocks, condensed in a series of reactions to form a long polyketide chain. The polyketide chain undergoes cyclization and dehydration to form Noranthrone, an early intermediate in the pathway. Noranthrone is further modified by enzymes to form versicolorin B through a series of oxidation, reduction, and cyclization reactions. Versicolorin B is converted to versicolorin A through additional enzymatic steps, including the action of cytochrome P450 monooxygenases. Versicolorin A is transformed into Sterigmatocystin, a critical intermediate in the aflatoxin biosynthesis pathway, through further enzymatic reactions involving ring closures and oxidation. Sterigmatocystin is methylated to form O-methylsterigmatocystin, another important intermediate. O-Methylsterigmatocystin is oxidized and undergoes additional modifications to form aflatoxin B1 (AFB1). AFB1 can be reduced to form aflatoxin B2 (AFB2). The conversion of O-methylsterigmatocystin to aflatoxin G1 (AFG1) involves similar steps to those forming AFB1, but includes additional hydroxylation and oxidation reactions. AFG1 can be reduced to form aflatoxin G2 (AFG2). The biosynthesis of aflatoxins is catalyzed by a series of enzymes, each responsible for specific steps in the pathway. Polyketide Synthase (PKS) initiates the synthesis by forming the polyketide chain. Cytochrome P450 Monooxygenases catalyze various oxidation steps, crucial for the formation of intermediates like Versicolorin A and Sterigmatocystin. Dehydratases and Reductases are involved in cyclization and reduction reactions, shaping the polyketide chain into cyclic intermediates. Methyltransferases catalyze the methylation of Sterigmatocystin to O-methylsterigmatocystin. Finally, Oxygenases participate in the final steps to form AFB1 and AFG1 (Caceres et al. 2020; Khan et al. 2021; Li et al. 2024a, b). Figure 1 shows steps of aflatoxin synthesis (Gacem & Ould El Hadj-Khelil 2016).

## Hazards of aflatoxin

These toxins are highly hazardous to both human and animal health, with aflatoxin B1 being the most toxic and carcinogenic. Aflatoxins are not only potent hepatotoxins and carcinogens but also pose risks to various other organ systems and health outcomes. Chronic exposure to aflatoxins, especially through contaminated food and environmental sources, can lead to a range of adverse health effects (Shan 2020).

### Carcinogenicity

Aflatoxin-induced carcinogenicity is a significant public health issue, particularly in developing countries with high exposure levels. Exposure to aflatoxins causes initial genetic damage. This can occur through the consumption of contaminated food, leading to DNA adduct formation and subsequent mutations in critical genes. Mutated cells with growth advantages proliferate more rapidly than normal cells. Chronic exposure to aflatoxins can provide a continuous stimulus for this clonal expansion. Over time, additional mutations accumulate, and the cells undergo further genetic and epigenetic changes. This leads to the development of malignant tumors, like hepatocellular carcinoma (HCC) (Gerdemann et al. 2023; Wang et al. 2023a, b, c). A study examining aflatoxin B1 (AFB1) metabolism in different species showed significant variations. Mice metabolized AFB1 rapidly, while rats and humans had lower rates. All species produced aflatoxin M1, with humans also producing aflatoxin Q1 and aflatoxicol. Rats had high DNA adduct formation, while mice had lower levels and faster repair. Protein adducts formed rapidly in all species (Gerdemann et al. 2023). Aflatoxin B1 can cause mutations during DNA replication by forming aflatoxin-DNA adducts which can lead to mutations, particularly G to T transversions, where a guanine base is erroneously replaced by thymine. The combination of viral hepatitis and aflatoxin exposure synergistically increases the likelihood of liver cancer development. Besides the health implications, aflatoxin contamination leads to significant economic losses due to crop destruction and reduced productivity. A study conducted by Yongdong Niu and colleagues demonstrated that the increased AFB1 hepatotoxicity by activating cytochrome p450 3A4 (CYP3A4) and decreasing levels of glutathione S-transferase Mu 1 (GSTM1) in cell lines (Niu et al. 2021).

### Acute toxicity

**Fig. 2:**
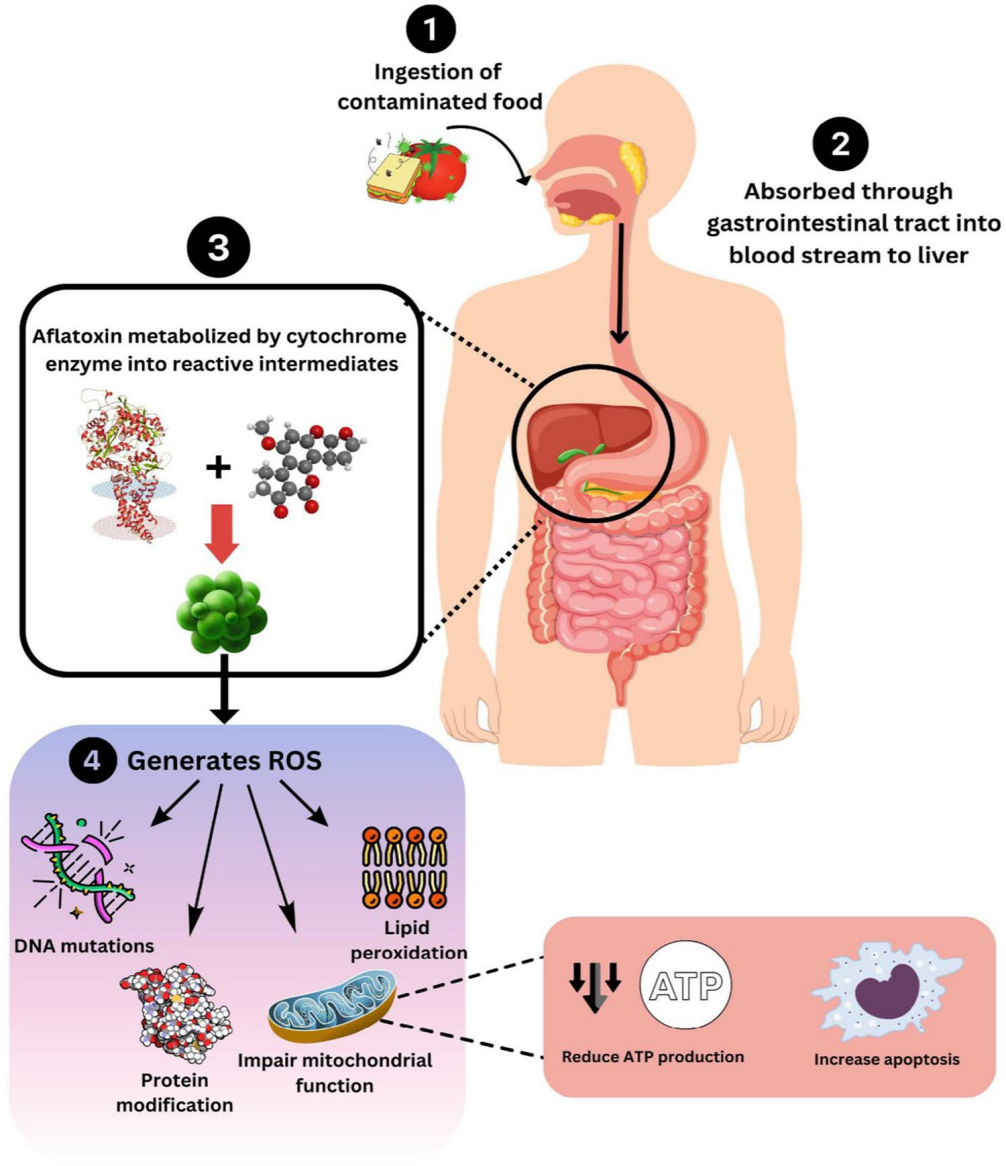
Acute toxicity caused by aflatoxin

Acute toxicity caused by aflatoxins can lead to severe and potentially fatal health effects, primarily affecting the liver. Acute aflatoxin toxicity typically occurs through ingestion of heavily contaminated food. Aflatoxins are absorbed from the gastrointestinal tract into the bloodstream. Once in the bloodstream, aflatoxins are transported to the liver, the primary site of detoxification and also the main target organ for toxicity (Abrehame et al. 2023). In the liver, aflatoxins are metabolized by cytochrome P450 enzymes into reactive intermediates, such as aflatoxin B1-8,9-epoxide (Cao et al. 2022). These reactive metabolites can bind to cellular macromolecules, including DNA, proteins, and lipids, leading to cellular damage. The reactive metabolites induce oxidative stress by generating reactive oxygen species (ROS), which cause lipid peroxidation, protein modification, and DNA damage. Aflatoxin metabolites can impair mitochondrial function, leading to reduced ATP production and increased apoptosis (Wang, et al. 2023a, b, c). Figure 2 shows a summary of the effects.

Acute exposure can cause aflatoxin-induced hepatitis, characterized by inflammation of the liver. Also, this results in jaundice, where the skin and eyes turn yellow due to elevated bilirubin levels. Severe exposure can lead to massive hepatic necrosis and liver failure. A case–control study in Eastern Ethiopia found that older age, farming occupation, family history of liver disease, hepatitis B infection, and high levels of aflatoxin B1 exposure were significantly associated with an increased risk of liver cirrhosis (Mekuria et al. 2023).

Aflatoxin poisoning can cause nausea, vomiting, abdominal pain, fluid retention, and bleeding disorders. In severe cases, it can lead to hepatic encephalopathy, characterized by confusion, altered consciousness, and coma (Alam et al. 2022; Richard et al. 2020).

### Immune system suppression

**Fig. 3:**
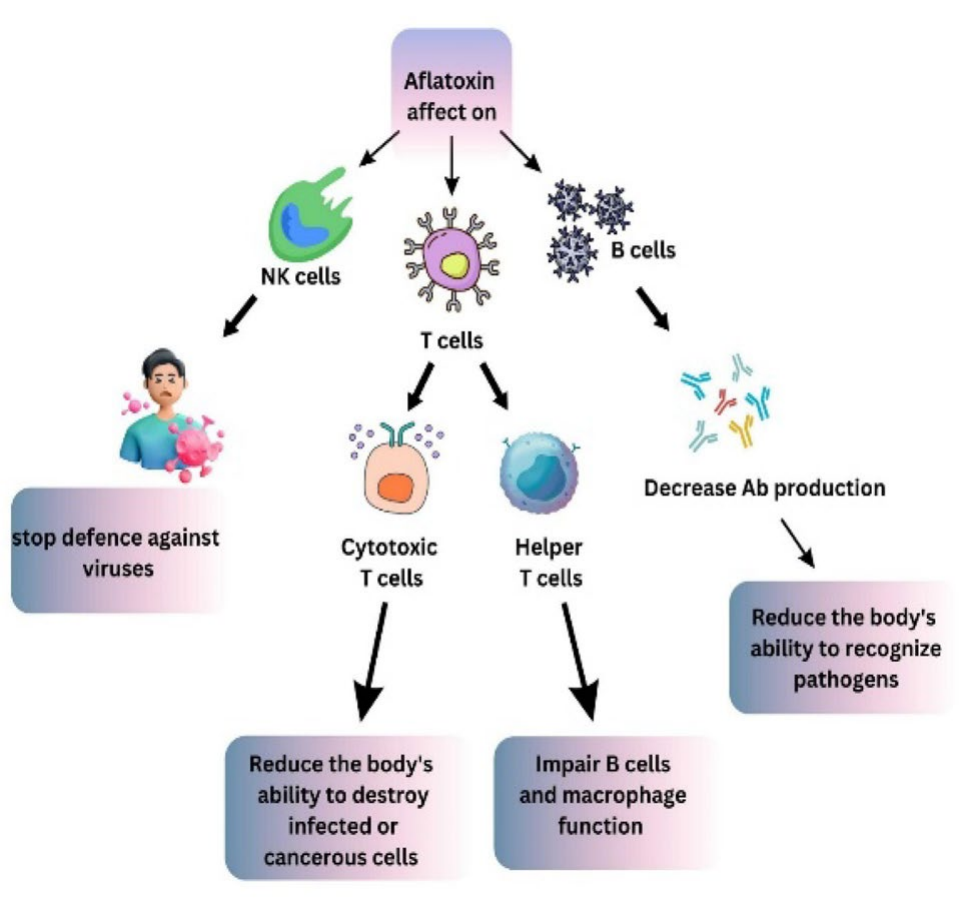
Aflatoxin causes immune system suppression

Aflatoxins, particularly aflatoxin B1, are known to suppress the immune system, making individuals more susceptible to infections and diseases. They directly affect B lymphocytes leading to reduced antibody production (Fig. 3). Aflatoxin can negatively impact T cells (both CD4 + and CD8 +), NK cells, and antigen-presenting cells, compromising the body's ability to fight infections and cancer (Saha Turna et al. 2023; Shan 2020). A study reported that exposure of pregnant and lactating C57Bl/6 J female mice to aflatoxin B1 (AFB1) at 400 μg/kg body weight/day, administered three times a week from embryonic day 11.5 to postnatal day 21, caused gut immunological changes in their offspring. These included an increase in intestinal lymphocyte numbers, reduced expression of microbial defense genes, and decreased cytokine production by intestinal type 2 innate lymphoid cells (ILC2). Although chemically induced colitis susceptibility was unaffected, the immune alterations were associated with gut microbiota changes and higher vulnerability to early-life *Eimeria vermiformis* infection. This indicates that maternal AFB1 exposure can impair intestinal barrier homeostasis in offspring, reducing their capacity to tackle intestinal pathogens (Bastos-Amador et al. 2023). Individuals exposed to aflatoxins are more prone to bacterial infections and increased risk of fungal and parasitic infections due to weakened humoral and cellular immunity. The suppression of T cell and NK cell function also increases the risk of viral infections and reduces the effectiveness of the immune response to viral challenges (Sun et al. 2023). In addition, The immune system's ability to respond to vaccines is compromised, leading to reduced vaccine efficacy and lower levels of protective antibodies (Kipkoech et al. 2023). While aflatoxins primarily cause immune suppression, the resulting immune imbalance can sometimes trigger autoimmune responses, where the immune system attacks the body's own tissues (Kraft et al. 2021). Persistent immune suppression and oxidative stress can lead to chronic inflammation, contributing to the development of various chronic diseases (Leyane et al. 2022).

### Economic impacts

Aflatoxins have far-reaching economic impacts that affect agriculture, trade, public health, and overall economic development, particularly in regions where aflatoxin contamination is prevalent. Aflatoxin contamination primarily affects staple crops such as maize, groundnuts (peanuts), and tree nuts. These crops are highly susceptible to infection by *Aspergillus* fungi, especially in warm and humid climate (Boni et al. 2021). A study in Tanzania found widespread aflatoxin contamination in groundnuts (92–100%) and maize (10–80%). Aflatoxin levels exceeded safety limits in both crops. The estimated daily intake of aflatoxin from maize significantly exceeded the safe limit for adults, raising concerns for public health in Tanzania (Boni et al. 2021).

International trade regulations impose strict limits on acceptable aflatoxin levels in food and feed products. The European Union, for instance, has some of the most stringent aflatoxin standards. to protect consumer health but can pose substantial barriers to trade for exporting countries, especially those in developing regions. Crops that exceed permissible aflatoxin limits are rejected by importing countries, resulting in financial losses for exporters and a loss of market access affecting the profitability of agricultural exporters impacts the entire supply chain, including processors, distributors, and retailers (Garcia‐Alvarez‐Coque et al. 2020).

### Food loss

Aflatoxin contamination significantly contributes to food loss and wastage across various stages of the food supply chain, from production to consumption. Understanding the mechanisms and consequences of aflatoxin-related food losses is crucial for addressing this pervasive issue. Aflatoxins are produced by fungi of the *Aspergillus* genus, primarily *Aspergillus flavus* and *Aspergillus parasiticus,* which thrive in warm and humid conditions. These fungi infect crops such as maize, peanuts, tree nuts, and other staples during pre-harvest, especially when crops are stressed due to drought or insect damage. Infected crops show visible signs of mould growth, discolouration, and an earthy or musty odour, rendering them unsuitable for human or animal consumption (Boni et al. 2021).

Improper harvesting and handling practices can exacerbate aflatoxin contamination. Delayed harvesting or inadequate drying of crops post-harvest can promote fungal growth and increase aflatoxin levels, further reducing the market value of the harvested produce (Xu et al. 2022a).

Aflatoxin contamination can persist and even amplify during food processing and storage. Food processing industries, such as milling, and food manufacturing, may reject or discard raw materials with high aflatoxin levels (Awuchi et al. 2021; Jallow et al. 2021). Contaminated crops can contaminate entire batches or lots of processed products, leading to financial losses and reduced production efficiency. Inadequate storage conditions, such as high humidity and poor ventilation, can accelerate aflatoxin production and contamination (Jallow et al. 2021; Liu et al. 2020). Stored grains and nuts are particularly vulnerable, as aflatoxin-producing fungi can proliferate in warm, damp environments, leading to spoilage and rendering the stored goods unfit for consumption or commercial use (Gong et al. 2024).

### Other health issues

Apart from liver damage and immune suppression, aflatoxins are associated with several other health issues, both acute and chronic, depending on the level and duration of exposure. Acute ingestion of aflatoxin-contaminated food can lead to gastrointestinal symptoms such as nausea, vomiting, and abdominal pain (Ajmal et al. 2022). It may cause diarrhea, which can exacerbate dehydration and electrolyte imbalances. Aflatoxin toxicity can impair blood clotting mechanisms, leading to hemorrhagic episodes and increased bleeding tendencies (Awuchi et al. 2020). Chronic exposure to aflatoxins may contribute to the development of anemia due to reduced synthesis of blood components in the bone marrow (Uluışık et al. 2020). Exposure during critical developmental stages, such as childhood and pregnancy, may impair growth and development in children, leading to stunted growth and reduced cognitive function. There is evidence suggesting that aflatoxin exposure during pregnancy may increase the risk of birth defects (Ismail et al. 2021). Aflatoxins can affect kidney function, leading to nephrotoxicity and chronic kidney disease (CKD) in severe cases. The mechanisms involve oxidative stress and inflammatory responses affecting renal tissue (El‐Mekkawy et al. 2020). Also, neurological symptoms occur such as headache, dizziness, and altered mental status, particularly in cases of acute aflatoxicosis (Awuchi et al. 2021). Respiratory Issues occur through inhalation of aflatoxin-contaminated dust or spores that may irritate the respiratory tract, leading to symptoms such as coughing, wheezing, and shortness of breath (Masciarelli et al. 2020). Chronic inflammation and oxidative stress induced by aflatoxin exposure may contribute to the development of hypertension and cardiovascular complications (Altyar et al. 2023). Aflatoxin exposure has been implicated in disrupting glucose metabolism and insulin sensitivity, potentially contributing to the development of metabolic syndrome and type 2 diabetes mellitus. A study involving 423 participants analyzed AFB1-albumin adduct levels concerning various metabolic conditions, including diabetes, obesity, central obesity, metabolic syndrome, and non-alcoholic fatty liver disease (NAFLD). Results showed a significant association between high AFB1-albumin adduct levels and diabetes (highest vs. lowest quartile POR = 3.74, 95% CI: 1.71–8.19; P-trend = 0.003), but no significant associations with the other conditions (Alvarez et al. 2022). Aflatoxins may interfere with endocrine function, affecting hormone production and regulation (Verga et al. 2024).

## Methods of detoxification of aflatoxins

AFs can be detoxified using physical, chemical, and biological detoxification methods, and a great deal of research has been carried out using these methods in the past few decades (Zhou et al. 2018). The long-term consumption of food contaminated with AFs can induce inflammatory damage to hepatocytes (Zhang et al. 2019). Furthermore, the AF-DNA adducts can result in the production of cancer cells, leading to liver cancer (Śliżewska et al. 2019). An increase in aflatoxins has been observed during the last few years, likely might be due to climatic change, which has stimulated the appearance of new combinations of mycotoxins/host plants/geographical areas and has enhanced the ability of *Aspergillus* species to produce aflatoxins (Wang et al. 2023a, b, c).

Aside from issues with food safety, contaminated crops cause the agriculture sector to suffer large annual financial losses (natural resource waste). This makes the crops hazardous for ingestion. Alternative pre- and post-harvest technologies are needed in this case to reduce the amount of contamination in commercial foods or, at the very least, guarantee that AF levels are below safe limits (Atakan & Caner 2021). Although there have been improvements in food safety and processing, the incidence of foodborne infections is on the rise. Ensuring the safety and security of food and agricultural products has emerged as a major challenge. The increasing demand for food, shortages in the food supply, and the widespread occurrence of food quality issues such as adulteration and fraud have led to concerns about food safety and nutritional insecurity (Cherif et al. 2023).

### Aflatoxins detoxification

#### Biological methods

Biological control techniques could be a useful tool in the current global context of climate change and rising mycotoxin hazards to help reduce extended background-level mycotoxin exposure. This is particularly true for certain farming methods, including organic farming, where the use of chemical antifungal agents is prohibited (Kos et al. 2023).

Various pre-harvest biological control methods have proven effective in reducing the harmful effects of AFs. These methods rely on the beneficial properties of different bacteria, yeasts, fungi, and their secretions, such as competitive exclusion or bio fungicide activity. As a result, several combined pre-harvest biocontrol technologies have been successfully developed and implemented. Biological detoxification approaches utilize certain bacteria that bind and/or convert AFs into less harmful chemicals (Peles et al. 2021). The purpose of this control method is to introduce non-harmful biological agents, such as bacteria or yeasts, in order to compete with pathogenic fungi for resources. This competition aims to decrease the growth of the fungal and its ability to produce mycotoxins. Non-aflatoxigenic strains of *Aspergillus spp.* are used as biocontrol agents to outcompete aflatoxigenic strains and inhibit their dominance, hence preventing the generation of aflatoxins (Daou et al. 2021).

##### Biocontrol

Many microorganisms live in the ecosystem, creating a complex microbial community (von Hertwig et al. 2020). *Aspergillus flavus* lives in soil, therefore soil ecotoxicology is becoming a safety concern. Due to soil complexity and heterogeneity, secondary metabolites such as AFs are difficult to study ecologically (Fouché et al. 2020). Thus, co-cultivation is a promising method for reducing contaminants in crops, animal feed, and the environment. Exploitation and interference competition occur between harmful and beneficial microbes (Sarrocco et al. 2019). Co-culturing *Aspergillus flavus* and *parasiticus* with *Salmonella* reduces colony size and spore production of both fungus and AF levels (AFB1, AFB2, AFG1, and AFG2) are also decreased. By secreting inhibitory compounds, *Aspergillus oryzae* and a non-aflatoxigenic *A. flavus* can limit AFB1 synthesis and proliferation. Transcriptome sequencing has shown that *AflS, FarB*, and *MtfA* are involved in AF biosynthesis. The products of the synthetic gene cluster and two transcription factors, BrlA and AbaA, were significantly down-regulated, which may lead to the down-regulation of conidia-specific genes such RodA and RodB (Yang et al. 2020). After 24 h of co-cultivation with *Aspergillus niger, Aspergillus flavus* growth was reduced, resulting in a 42.8% drop in AFB1 production. Later studies found that when bacteria are cultivated together, their biological processes may cause genetic alterations or awaken latent gene clusters, reducing AF production (Guan et al. 2021).

A study by Ali et al. investigated the use of Coumarin, a structural component of aflatoxins, as a screening tool to identify bacteria capable of degrading these mycotoxins. Screening on coumarin media led to the isolation of 10 bacterial strains, including species of *Bacillus*, *Pseudomonas*, *Enterococcus*, and *Stenotrophomonas*. Notably, *Pseudomonas fluorescens* SZ1 exhibited exceptional aflatoxin degradation capabilities, effectively degrading all four major aflatoxin types with high efficiency and speed. Further analysis revealed that factors such as pH and the presence of certain metal ions significantly influenced the degradation process, suggesting the involvement of enzymatic reactions (Ali et al. 2021). A bacterial laccase enzyme, CotA, has been shown to directly oxidize AFB1 into less toxic metabolites, namely aflatoxin Q1 (AFQ1) and epi-AFQ1, without the need for additional redox mediators. Laccases are enzymes with the potential to degrade aflatoxin B1 (AFB1) by catalyzing its oxidation. This finding highlights the potential of utilizing bacterial laccases as a promising strategy for the enzymatic detoxification of aflatoxins (Subagia et al. 2024). Another bacterium *Rhodococcus erythropolis* has been shown to possess the ability to biodegrade aflatoxin B1 (AFB1), by producing extracellular enzymes that can effectively degrade AFB1, significantly reducing its toxicity as demonstrated by a decrease in mutagenicity (Alberts et al. 2006).

##### Fermentation

Aflatoxin exposure makes food safety difficult, posing health dangers. Listed as a Group 1 human carcinogen by the International Agency for Research on Cancer, aflatoxin B1 is highly mutagenic and carcinogenic (Kumar et al. 2022). LAB strains as fermentation starters could form the basis of a well-established and widely available technology that could make nutritious and safer food more accessible in low-income nations free of mycotoxin contamination (Sionek et al. 2023). Fermentation has preserved food for millennia, and numerous traditional fermented items are available globally. Plant-based fermented meals are safe and effective thanks to lactic acid bacteria (LAB). The nutritional content, sensory qualities, shelf life, and safety of food products depend on food-fermenting LAB metabolism (Şanlier et al. 2019). Due to their GRAS status, most LABs and LAB-fermented foods are accepted by consumers as natural and useful foods. *Lactiplantibacillus plantarum* fermentation starter cultures are long-standing starting cultures in milk, meat, and vegetables. Sauerkraut, sourdough, pickles, and brined olives also contain them (Behera et al. 2018). Many strains of lactic acid bacteria (LAB) have been used as antifungal and anti-mycotoxigenic medicines because they inhibit fungi and absorb their toxins (Sadiq et al. 2019). *L. plantarum,* as a starting culture in the fermentation of a protein-rich plant-based food product, significantly reduced free aflatoxin B1 up to 90%. Live or heat-inactivated *L. plantarum* cells grown with free aflatoxin B1 for varied durations produced similar results in vitro. By fermenting fava bean suspension, aflatoxin B1 was eliminated at a high rate, emulating food preservation (Rämö et al. 2022).

#### Chemical methods

Presently, the selection of chemical approaches for reducing AF typically involves the use of different chemical agents, ozonation, and adsorption. Nevertheless, several novel chemistry-based techniques are currently being developed, while acids like citric, lactic, tartaric, and propionic have shown promising aflatoxin-reducing properties, others, such as succinic, acetic, ascorbic, and formic acids, have demonstrated limited efficacy (Jubeen et al. 2020; Rushing & Selim 2019).

##### Ammoniation

Physical, chemical, and biological treatments have been studied to reduce aflatoxin levels in cereal grains and oilseed meals. According to the literature, the most common method, which the applicant uses, involves subjecting mycotoxin-contaminated animal feed to ammonia at high temperature and pressure and evaporating the ammonia. Aflatoxin levels drop by 96% to 99% with this method. Based on this and previous research, numerous nations use ammoniation to cleanse polluted diets (Chain (CONTAM) et al. 2021). No ammoniation process is universally accepted, instead ammonia levels, temperature, pressure, water content, exposure period, initial contamination level, and substrate material affect procedure performance. Ammonia breaks the AFB1 lactone ring and this creates an ammonium salt with hydroxy acid, reducing toxicity.

The majority of AFB1 detoxification investigations uses aqua-ammonia or NH4OH. However, ammonia gas detoxification of AFB1 is little studied. ammoniated diets (Zhang et al. 2022a, b). A high-pressure and high-temperature (HP/HT) or atmospheric pressure and ambient temperature technique uses anhydrous ammonia and water to treat the contaminated product in a controlled setting.

##### Ozonation

A strong oxidizer, ozone (O_3_) is considered safe in the food business as an antimicrobial agent. Ozone oxidizes enzyme sulfhydryl and amino acid groups or targets cell wall polyunsaturated fatty acids to affect fungal cells (Sharma et al. 2021). Mycotoxin-producing Fusarium is most affected by ozonation. Ozonation also affects *Aspergillus* and *Penicillium*, but to a lesser extent. Research shows that O_3_ gas may entirely kill *Fusarium* and *Aspergillus*. Ozone fumigation reduces spore germination and toxin production. It is believed that ozone interacts with the functional groups inside mycotoxin molecules to change their molecular structures and produce products with lower molecular weight, fewer double bonds, and lower toxicity (Afsah-Hejri et al. 2020). UV irradiation, oxygen electrical discharge, and water electrolysis generate ozone. Ozone's sudden breakdown without leaving residues makes it a promising food processing ingredient (Guo et al. 2021). It reacts strongly with unsaturated carbon double bonds (C = C) and helps in oxidation. The breakdown of tagged AFB1 with O_3_ produced 3-ketones, organic acids, and volatile chemicals or mineral products such CO_2_, O_2_, and H_2_O. Ozone (O_3_) at concentrations of 5, 7.5, and 9 mg per liter on almonds, pistachios, and peanuts for 140 and 420 min at 70% relative humidity reduced AFB1 by 23% (Babaee et al. 2022). To conclude, ozone can kill viruses and break down their byproducts without leaving ozone residue. At room temperature and neutral pH, this reaction happens swiftly. Powerful oxidizer ozone attacks aflatoxin's furan ring's 8, 9 position double bond electrophilically and primary ozonides rearrange into monozonide derivatives such as aldehydes, ketones, and organic acids (Somda et al. 2023).

##### Chemical binding agents

To mitigate the adverse impacts of mycotoxins in animal feeds, one approach is to incorporate toxin-sequestering agents into the feeds (Holanda and Kim 2021). The addition of mycotoxin binders to diets contaminated with mycotoxins resulted in enhanced growth performance and increased nutritional digestibility in pigs (Clarke et al. 2018). Nevertheless, the impact of toxin-sequestering agents is not consistent and can vary based on the specific toxin binders, target mycotoxins, and animal species involved (Holanda and Kim 2021). Various compounds that can sequester mycotoxins have been tested and are currently being traded globally. Nevertheless, only a limited number of them have undergone thorough examination to substantiate their efficacy as mycotoxin binders. Activated carbons, certain clays, and an esterified glucomannan produced from yeast cell walls have been utilized for in vitro experiments to measure the binding of AFB1 (Hojati et al. 2021).

#### Physical methods

Aflatoxins are chemically stable molecules that have breakdown temperatures ranging from 237 to 306 °C, which means they are not eliminated by normal thermal processing or cooking. Regrettably, there is no singular measure that can be employed to completely prevent or eliminate mycotoxin contamination (Marshall et al. 2020). However, there exist a variety of physical control measures that can be implemented and have been widely evaluated, therefore several recommended measures have been found useful in reducing AF levels in this review (Sipos et al. 2021).

##### Sorting and cleaning

Dust, husks, mould-contaminated materials, mechanical sorting, and washing are all part of cleaning. Tiny, shriveled, moldy, discoloured, and damaged seeds cause 80% of aflatoxin contaminations. Washing seldom removes aflatoxins (AFs) due to their low water solubility. However, eliminating aflatoxin from food's interior structure is difficult (Somda et al. 2023). Numerous studies have shown that cleaning and sorting cereals eliminates contaminated grains and significantly reduces mycotoxins. The initial contamination level in the raw material and the fraction of materials removed after cleaning greatly affect the cleaning process's performance (Schaarschmidt & Fauhl-Hassek 2018). The most dangerous mycotoxins that can infect maize are aflatoxins (AFs), which also affect humans and animals (Xu et al. 2022b). Removing damaged and infected grains from maize reduced aflatoxin levels by 40% to 80%. Furthermore, carefully sorting broken and damaged kernels reduced maize fumonisins by 84%, and it reduced AFs and fumonisins in white maize by 94–95%. Using UV light at 365 nm, fluorescence-based manual sorting reduces aflatoxin contamination in dried figs and peanuts by removing luminous substances however, this method is rarely used in practice (Pascale et al. 2020).

##### Irradiation

UV light makes *A. flavus*-contaminated corn kernels glow greenish-yellow, making separation straightforward. However, this reaction may not always occur, and internal fungal contaminations may not have visible effects (Sipos et al. 2021). Another method uses red and green light reflectance to distinguish peanuts with AF (Tao et al. 2018). A cost-effective multi-spectral analyzer was developed to study maize kernels at nine wavelengths (λ = 470–1550 nm). Pulsed high-power ultrasonic radiation caused cavitation, structural changes, protein and enzyme inactivation. Another study indicated that pulsed ultrasound at 1.65 W/cm3 for 10 min reduced AFB1 in maize flour slurry with treatment clearance of 11% (Liu et al. 2019). Different AFs have different absorption peaks and UV radiation and oxygen destroy AFs. AFB1 absorbs most at 223, 265, and 362 nm, AFB2 at 265 and 363 nm, and AFG1 at 243, 257, 264, and 362 (Kumar 2018). When added to pure water and subjected to UV radiation, AFG1, AFB1, and AFB2 concentrations were reduced to 67.22%, 98.25%, and 29.77% respectively (Patras et al. 2017). Gamma irradiation reduces AFB1 in naturally contaminated corn kernels and mycotoxins can be reduced by 69.8% to 94.5% with 1–10 kGy irradiation (Serra et al. 2018). Reduced soybean AFB1 was found to be reduced at doses over 10 kGy (Zhang et al. 2018) and over 95% was reduced by the combined irradiations (Patil et al. 2019).

##### Adsorption

Another effective, eco-friendly, and modern technology, like adsorption is used since AFB1 is one of the biggest hazards to food safety and the environment and has strong chemical and thermal stability (Zhang et al. 2022a, b). Few studies have examined the adsorption removal of aflatoxins from foods, and the types of aflatoxins and foods involved were limited. We have only found reports of AFB1 removal from apple juice or edible oil. An external magnetic field separates the adsorbents from the edible oil, complicating the operation. Thus, innovative adsorbents that are easy to use, suited for a variety of foods, and capable of removing multiple aflatoxins must be developed (Dai et al. 2022). The polydopamine (PDA) covering is biocompatible, homogenous, and may conjugate with amino-containing compounds via Michael addition and Schiff-base reaction due to its quinone functional groups. PEI, a charged polymer with several amine groups, is good at separating and purifying pollutants and biologics. A mussel-inspired membrane adsorber was created by modifying the polyvinylidene fluoride (PVDF) microfiltration membrane with PDA and PEI. PEI molecules interacted with AFB1 to remove this mycotoxin and an alkaline solution was employed to regenerate and degrade the membrane (Li et al. 2018).

##### Photocatalysis

Another method that can be employed to eliminate mycotoxins is the photocatalytic detoxification of mycotoxins in food (Magzoub et al. 2019). This technology is considered non-thermal and emergent. This process involves a chemical reaction that is triggered by the absorption of photons by a solid photocatalyst. This reaction leads to oxidation/reduction reactions on the surface of the photocatalytic material, resulting in the creation of free radicals. These free radicals then interact with contaminants, such as mycotoxins, and help break them down or convert them into less harmful compounds through oxidation reactions (Murugesan et al. 2021). Several safety concerns in the agricultural and food industries encompass enduring pollution, decay, infestation, pollution from agricultural chemicals, and contamination from antibiotics (Jiang et al. 2022). The majority of conventional mycotoxin degrading technologies have evident drawbacks. Although surface pollution can be controlled, these methods may lead to food safety issues, including alterations in nutritional or sensory characteristics (such as taste, colour, and texture), inadequate effectiveness, and potential financial consequences (Li et al. 2024a, b).

Biological approaches, such as those used to detect toxins, are often very specific and cannot be simultaneously applied to many poisons. Chemical techniques might potentially harm the quality of food, while many physical techniques are costly (Zhi et al. 2023). Hence, there is a requirement for novel approaches to mitigate risks in the food and agriculture sectors, as well as in healthcare. Furthermore, food manufacturers are seeking methods to diminish or decrease allergies, either in food products or during the manufacturing process, as the safety of foods concerning their immunological reactivity is becoming more significant (Jiang et al. 2022). Various ways are now under investigation, and novel technologies are being created. Hydrostatic pressure, ultrasound, pulsed electric fields, and pulsed light are among the several technologies available (Zadeh 2023).

##### Plasma classifications and the distinctive features of cold plasma

Plasma is considered as the fourth state of matter. It is formed by ionizing a neutral gas composed of free radicals, ions, zero net electrical charge molecules, and excited and non-excited atoms (Ozen & Singh 2020). The ionization energy needed can be obtained by heat, microwaves, radio waves, and electricity. Plasma can be classified in different ways which, either present in nature by itself or artificially synthesized (S. Sharma & Singh 2020). Based on thermal conditions, plasma can be classified into thermal, non-thermal, and local thermal equilibriums (Ozen & Singh 2020). The non-thermal plasma, where the temperature of ions and nonionized species is around room temperature but the temperature of electrons is significantly high, is termed as Cold Plasma (CP) (Sharma & Singh 2020). Also based on pressure employed during plasma application: plasma is classified as low-pressure and atmospheric pressure plasma. Many atmospheric pressure plasma generation technologies are used for food processing applications. Some of these technologies are direct barrier discharge (DBD), microwave (MW), and atmospheric pressure plasma jets (APPJ). The mechanism of action of all these technologies to generate plasma is initiating and sustaining electron collision under their specified conditions, such as photoionization, radio frequency, and gas discharge (Ozen & Singh 2020). DBD and plasma jet are plasma sources that are most commonly used for food research due to their commercial availability and simple construction (S. Sharma & Singh 2020). DBD plasma is composed of double electrodes, at least one of them covered with a selected dielectric barrier material so that this type of technology provides stability and uniformity in the decontamination and treatment of samples by performing in a large area. APPJ includes a double concentric electrode embedded in a nozzle through which the carrier gas passes (Ozen & Singh 2020). In this article, we discuss cold plasma (CP) or non-thermal plasma, which is a novel exploration processing technique with multiple applications in various industries. In the food industry, it can range from functional modification, and microbial decontamination, to enzyme inactivation. Due to its economical advantage of using atmospheric air as a gas, this technology can be performed at atmospheric pressure and ambient temperature (Sruthi et al. 2022). CP can be generated either by atmospheric pressure, therefore termed atmospheric CP (ACP), or low pressure, according to generation conditions, which both generate the same electron density range and similar reactive species (Bourke et al. 2017). Another type of CP is called high voltage atmospheric cold plasma (HVACP), although not recently recognized. Its efficacy in different applications has been observed. Despite its benefits, in other situations, it has side effects like inducing undesirable deformities such as oxidation of lipids in oily foods (Olatunde et al. 2019). Also, this technology has the benefit of limited modification of food components with a significant role in biological contamination. One of the main advantages of this technology is that it can be performed at room temperature without chemicals or enzymes added, and the treatment may span minutes or even seconds rather than hours (Ott et al. 2021). In general, CP has proved to be effective against the majority of food-borne pathogenic microbes, enzyme inactivation, and toxin removal (Liu et al. 2023). Atmospheric pressure cold plasma (APCP) also is an emerging technology being explored for the treatment of aflatoxins. APCP operates at room temperature and atmospheric pressure, generating a mixture of reactive species such as ions, electrons, and radicals that can effectively break down aflatoxins without the need for extreme conditions or harmful chemicals. In the context of aflatoxin treatment, APCP is applied directly to contaminated food. The reactive species generated by the cold plasma interact with the aflatoxin molecules, leading to their degradation and reducing their toxicity. This process is particularly advantageous because it can be conducted at low temperatures, preserving the quality and nutritional value of the treated products. APCP requires longer exposure times. A summary comparing the 3 different treatment modalities with CP is shown in Table 2 (Yepez et al. 2022).

**Table 2:** Comparison of plasma methods for aflatoxin treatment

Aspect	Cold Plasma (CP)	Atmospheric Pressure Cold Plasma (APCP)	High Voltage Atmospheric Cold Plasma (HVACP)	Dielectric Barrier Discharge (DBD)
General Overview	Non-thermal plasma technology for various applications. Operates at low temperatures making it suitable for treating heat-sensitive materials like food products	Plasma operates at atmospheric pressure, eliminating the need for vacuum systems	High voltage (tens of kV) plasma, generating a strong electric field	Plasma generated between two electrodes with a dielectric barrier
Aflatoxin Treatment	Effectively generates ROS and other reactive species that can degrade aflatoxins. However, its effectiveness can vary depending on the specific setup, gas composition, and treatment parameters	Effective in reducing aflatoxin levels, efficiency varies with gas, power, and duration	Highly effective, achieves significant reductions in short treatment times	Effective, depends on voltage, frequency, and dielectric material
Advantages	Preserves food quality, operates at low temperatures, adaptable	Versatile, scalable, easier to implement in industrial settings	High efficiency, shorter treatment times, robust results	Precise control, uniform treatment, minimal impact on food quality
Limitations	May require longer treatment times or multiple cycles for effective degradation	May require longer exposure times than HVACP or DBD for similar efficacy The reactive gas species leads to questions about safety, active life, and environmental impact	Can affect food quality, requires sophisticated equipment and safety measures	Complex setup, requires optimization for different food matrices
Best For	General applications, where food quality is the main concern	Industrial use, balancing effectiveness and ease of implementation	Highest efficacy in aflatoxin degradation, especially in research settings	Research and precision applications requiring uniform treatment

## Chemical mechanisms of aflatoxin degradation by cold plasma

Cold plasma treatment significantly alters aflatoxin B1's structure by generating reactive species like ozone, hydroxyl radicals, and superoxide radicals. These species interact with the aflatoxin molecule, leading to chemical modifications such as oxidation, epoxidation, ring cleavage, and addition reactions. These structural changes ultimately reduce the toxicity of aflatoxin B1, making cold plasma a promising technology for decontaminating food products. The aflatoxin occurring in the food matrix is subjected to degradation by reactive species including O, O_3_, OH, NO, and NO_2_ generated by the cold plasma. The generated reactive species bombard the chemical bonds of the aflatoxin molecules causing degradation or changing them into other harmless products (Apalangya 2024).

Utilizing advanced analytical techniques like HPLC and mass spectrometry, researchers observed significant AFB1 degradation after 5 min of HVACP (Humidified air cold plasma) treatment. Six major degradation products were identified, resulting from modifications to the AFB1 structure, particularly in the furofuran ring, cyclopentenone, and methoxy group. Two aflatoxin degradation pathways were proposed by Shi et al. when aflatoxin was treated with cold plasma. The first pathway involves a series of addition reactions, including hydration, hydrogenation, and the addition of aldehyde groups to the AFB1 molecule, driven by the activity of reactive species generated by HVACP, such as hydrogen atoms and hydroxyl radicals. The second AFB1 degradation pathway under HVACP primarily involves epoxidation and oxidation reactions. Epoxidation, likely mediated by hydroperoxyl radicals (HO_2_•), occurs at the terminal double bond of AFB1. Subsequently, the furofuran ring of AFB1 undergoes cleavage, leading to the formation of initial degradation products. Further oxidation reactions, involving a combination of reactive oxygen species such as hydroxyl radicals (OH), hydrogen peroxide (H_2_O_2_), and ozone (O_3_), contribute to the formation of subsequent degradation products (Shi et al. 2017a, b).

In another study, Shi et al. investigated the effectiveness of cold plasma treatment in degrading aflatoxins (AFs) in corn. Their study examined the influence of various parameters, including plasma exposure time, carrier gas type (air vs. a modified gas mixture), and relative humidity (RH). Results showed that a modified gas mixture (rich in oxygen) and higher RH levels significantly enhanced AF’s degradation, likely due to increased ozone generation during plasma treatment. Moreover, longer plasma exposure times led to greater AF’s reduction, with 62% and 82% degradation observed after 1 and 10 min of treatment at 40% RH, respectively (Shi et al. 2017a, b).

## Impacts of cold plasma treatment

### Impact of cold plasma on microorganisms

Among all the challenges facing the food, clinical, and healthcare sectors, microorganisms are the most powerful issue. Their resistance to broad-spectrum antibiotics and several biocidal agents encourages researchers to discover novel antimicrobial technologies. Pathogenic bacteria have been demonstrated as a major food safety issue, in addition to their toxins, viruses, mycotoxins, and pesticide residues (Bourke et al. 2017). Also, fungal infections in the food industry are a severe problem, not only for spoilage conditions but also for the secretion of mycotoxins, which can be carcinogenic, toxigenic, and mutagenic in humans. In addition to their production of hardy spores and thermostable toxins, which result in deleterious modifications and deformities of host metabolism (Ott et al. 2021). It is found that high-temperature sterilization can impact the original flavour, texture, colour, and other substrate characteristics for aquatic products. Therefore, CP or non-thermal decontamination technologies play a significant role as antimicrobial and fungicidal agents with prolonged food shelf life (Xu et al. 2023). It is shown that with an increase in the exposure time of cold plasma to samples, the antimicrobial activity increased. The content of reactive radicals, molecules, and atoms also increased, as these reactive components result in lipid peroxidation, enzyme inactivation, and DNA degradation, causing microbial inactivation (Yarabbi et al. 2023). A study revealed that treatment with HVACP significantly reduced the visible colony number of *Pseudomonas aeruginosa* with post-treatment time, which is prevented DNA replication, transcription, and translation and disrupted the system of protein secretion. In addition to that, it inhibited biofilm formation and damaged cell metabolic energy (Liu et al. 2022). Another study reported that a combination of HVACP and cinnamaldehyde could significantly enhance the killing effect of HVACP against *Salmonella enterica* and *Escherichia coli* (Lewis et al. 2024). It is proven that increasing cold plasma exposure time increases the inhibition of *Penicillium italicum* and decreasing in *Salmonella, E. coli*, and *Listeria*. This reduction can be attributed to reactive nitrogen and oxygen species generated in the cold plasma process. By using a scanning electron microscope (SEM), the structure of bacterial cells was seen to be damaged, with cellular leakage, and DNA damage (Yarabbi et al. 2023). It is also shown that hydroxyl radicals (OH) affect the cell membrane of microorganisms, which destroy membrane-related proteins. In addition to the reduction of a general number of *Gibberella fujikuroi* colonies at atmospheric pressure, it was proved that non-thermal plasma treated with (DBD) has significant antimicrobial activity against *Gibberella fujikuroi* (Pańka et al. 2022). In summary, the use of cold plasma technology as an antimicrobial agent is highly expanding because of its low cost, preservation of substances and nutrients, lack of environmental pollution, and significant reduction of microbial contamination (Ahangari et al. 2021).

### Blocking aflatoxin production with cold plasma treatment

Many studies and methods have been observed to treat aflatoxins but with limited success. Aflatoxin can resist the common traditional thermal tests applied in the food sector. Ultra Violet (UV) radiation is used as an aflatoxin-degrading method, but because of its low penetration depth and the preservation of residual toxicity by degradation, it is considered to have limited scope. Recently, CP is considered as a vital alternative for aflatoxin degradation in the food industry (Nishimwe et al. 2021). Certain studies revealed that total aflatoxin content is reduced by 50% for only 20 min under air plasma treatment (APT)). Another study reported that colonization of *Aspergillus flavus* on agar was most inhibited after treatment with a cold atmospheric plasma jet (Wu et al. 2021). Aflatoxin B1 (AFB1) is the severest and most toxic type among the four various types of aflatoxin (Nishimwe et al. 2021). It is found that by using HVACP treatment, AFB1 is degraded rapidly in corn. In addition to that, a significant study stated that treatment with nitrogen gas plasma highly degraded AFB1 and reduced its physiological activities (Wu et al. 2021). According to a recent study, the viable spore population of *A. flavus* is highly reduced by increasing the exposure time of plasma treatment as remarkable changes were found in the structure of hyphae in *A. flavus* (Makari et al. 2021). Low-pressure and atmospheric pressure plasmas have been reported to show a significant ability to reduce four major pure aflatoxins of hazelnuts, in addition to retaining their sensory attributes (Wu et al. 2021). Recent studies have reported a 46% reduction in AFB1 levels in wheat and 47% in rice by treatment of corona discharge plasma jets (CDPJs). Also, by applying HVACP therapy, a 66% reduction in AFB1 content in maize kernels (Rahnavard et al. 2024). It was observed that CP treatment can remove AFB1, aflatoxin B2 (AFB2), and aflatoxin G1 (AFG1) from peanuts. Similar studies showed the efficiency of cold plasma in the total aflatoxin content reduction of cobnuts. Also, this kind of plasma treatment can effectively reduce the concentrations of mycotoxins in date palms (Gavahian & Cullen 2020). Recently, a study proved the complete inactivation of *Aspergillus parasiticus* and *Aspergillus flavus* spores with the treatment of cold plasma, when artificially inoculated in groundnuts. That same study stated that after treatment with CP, a 95% reduction in AFB1 from *Aspergillus parasiticus* and about 96.8% reduction in AFB1 from *Aspergillus flavus* had been achieved (Devi et al. 2017). Despite the beneficial characteristics of plasma technologies in the degradation of aflatoxins, current research should discuss more about the safety aspects (Gavahian & Cullen 2020). Aflatoxin decontamination remains a vital challenge for the food industry, the efficiency of degradation depends on many parameters, including decontamination conditions, decontamination technology, and even food conditions (Pankaj et al. 2018a, b).

### Application of cold plasma technology on plants

Agriculture with low content of fertilizers and chemicals has been widely attracting attention. This challenge requires massive research and development. Therefore, a promising non-chemical technology must be explored. It is found that CP possesses several advantages related to agriculture, such as safety and vitality to seeds, crops, humans, and the environment as an ecosystem. Also, it offers short processing times and low operating temperatures (Pańka et al. 2022). Due to the action of plasma it modifies the chemical characteristics of liquid and transform it into mixtures of reactive oxygen and nitrogen species (RONS), including NO and H_2_O_2_ biomolecules, which can be considered as elements regulating plant metabolism and its responses to various stresses and also considered as signaling elements In many processes of cell machinery, authors have shown that plasma-activated liquids possess several properties that support the plant growth and development (Kocira et al. 2022). It is reported that treatment with CP in tomatoes, can accumulate the root and shoot biomass, enhance the root activity and increase the area of leaves, which results in increased nitrogen and phosphorus uptake (J. Jiang et al. 2018). It has also been reported the ability of CP to modify the seed surface which increases the water absorption of the seed, in addition to the generation of reactive oxygen species (ROS) and charged particles, CP treatment is capable of making cracks in the seed coat to facilitate water imbibition and prevent seed dormancy. A study revealed that treatment with CP for 5 min perfectly enhanced nutrient uptake and increased chlorophyll pigment content (Rasooli et al. 2021). In another study, by ameliorating the activity and morphology of centipede grass, CP treatment can improve phosphorus, nitrogen, and potassium uptake. Also, the same study found that soluble sugar content was increased. In addition, protease, and α-amylase activities were also improved after the CP treatment (Li et al. 2021). Multiple studies have shown that zinc-oxide nanoparticles (ZnONPs) have an impact on the absorption of heavy metals by fruiting plants and leafy vegetables. Heavy metals uptake was highly reduced in Soybean (Glycine max) treated with ACP in the presence of ZnONPs (Mahanta et al. 2022). In addition, the presence of nitrate, nitrite, and nitrogen oxide in cold plasma, can induce germination and break dormancy. It is also reported that atmospheric or air plasma with N2 modified the seed surface making it hydrophilic, therefore water uptake by surface wettability of seed increased. Also, oxidative stress generated due to ROS in the presence of plasma was able to induce biomass dry matter concentration, plant growth, and germination rate (Paatre Shashikanthalu et al. 2020). It is also proved that rice seeds treated with cold plasma were sterilized from seed-borne pathogens and yielded healthy emerging seedlings. In addition to that, treatment of cold plasma by DBD with an argon/oxygen mixture significantly reduced bacteria and fungi survival on ginseng seeds surface (Lee et al. 2021). Recently, a study reported that treatment with CP was capable of stimulating the defense system to produce metabolites related to stress conditions, such as salicylic acid, jasmonic acid, and glutamic acid, which improved plant resistance against stress (Liu et al. 2023).

### Application of cold plasma on food

#### Effect on carbohydrates

Carbohydrates contain oxygen, carbon, and hydrogen which are found in a variety of food products (Saremnezhad et al. 2021). They are a major compound in food and play a vital role in maintaining its quality. After treatment of cashew apple juice with CP, an increase in sucrose content after long exposure time was observed due to degradation of oligosaccharides with significant degree of polymerization, and degradation of all reducing sugars, such as glucose and fructose. Similar results have been reported after treatment of prebiotic orange juice. Authors suggested that the changes observed was due to the degradation and cleavage of glycoside bonds leading to macromolecule de-polymerization and oxidation of functional groups to maintain carboxyl, carbonyl compounds, hydroperoxides, lactones, and CO_2_ (Pankaj et al. 2018a, b). It was also revealed that the oxidation of hydroxyl functional groups to gluconic acid and 2-deoxygluconolactone, is referred to as the free radicals produced during irradiation of CP treatment (Bayati et al. 2024). Starch plays an important role in many formulations in food industry, among carbohydrates and treatment with CP can influence its thermal characteristics, swelling capacity, crystallinity, digestibility, solubility, and structure. Plasma treatment also can reduce the amylose content. In addition to that, it was found that starch treated with plasma, became more accessible to amylase and hydrolysis enzymes, because plasma was able to damage the interior and surface of starch granules besides starch de-polymerization (Saremnezhad et al. 2021).

#### Effects on lipids

Lipids are consisted of saturated, either monounsaturated or polyunsaturated, and non-saturated fatty acids. Lipids in food undergo an oxidation process in the presence of catalytic systems, such as heat, light, and metals. When ROS from plasma interacts with food lipids, the oxidation process is induced. It is reported that the major targets of ROS in lipid are methyl groups, with higher affinity for those linked by double bonds (Gavahian et al. 2018). Recent studies revealed a reduction in the C18:0 content with an increase in the short-chain fatty acids (SCFAs) (C10:0 and C12:0) in food treated with CP. It is also reported that an increase in the value of primary and secondary markers of lipid oxidation, such as hydroperoxide and n-hexanal, is an indication of significant role of CP in lipid oxidation of wheat flour (Saremnezhad et al. 2021). Another study found that the cell membrane-associated unsaturated fatty acids (UFA) (C16:1, C18:1 and C18:2) of *Salmonella typhimurium* are reduced significantly. It is reported that this reduction in UFA, is an indication of oxidative pressure generated from exogenous ROS, therefore it resulted in oxidative damage in the cell membrane of *S. typhimurium* (Lv & Cheng 2022).

#### Effect on protein and enzymes

Proteins are considered as a major functional and structural component of many foods, due to their high nutritional value and other properties. Any alteration to the amino acids which build up the protein complex, affect the spatial structure of protein and its functionality. Exposure of protein to plasma results in oxidation of amino acids with the generation of ROS. It is proved that the oxidation of amino acids leads to the formation of cross-links, fragmentation, unfolding, and conformation changes in the protein (Saremnezhad et al. 2021). A significant study suggested that cysteine, methionine, and aromatic amino acids content, was reduced after plasma treatment. In addition to that exposure to DBD resulted in RNase A inactivation by forming methionine sulfoxide. It is also proposed that aromatic amino acids, such as tryptophan, which are sensitive to oxidation, can be attacked by ROS, such as OH radicals or atomic oxygen (Tolouie et al. 2018). Recent study revealed that the interaction of amino acids, secondary structures and plasma reactive species, resulted in protein denaturation due to loss of α-helix and β-sheet. Another study reported changes in the structure of protein of wheat flour, due to sulfhydryl group oxidation and disulphide bond formation (Pankaj et al. 2018a, b). Enzymes are considered as biocatalysts that can influence the main groups of functional biomolecules present in food (Tolouie et al. 2018). The structure of enzymes can be changed by active plasma species by cleavage of chemical bonds and side-chain modifications. Many studies reported an increase of some enzyme activities after plasma treatment, such as amylase, phytase, and protease activities in mung beans. Another study documented that treatment with helium glow discharge plasma jet improved the activity of lipase enzymes. On the other hand, authors reported the loss of lactate dehydrogenase (LDH) activity after direct treatment with plasma due to the presence of long and short-lived reactive plasma species and UV photons related to plasma treatment (Saremnezhad et al. 2021). It was shown that the enzymatic activity of the alpha-amylase enzyme was improved in the germinating brown rice after cold plasma treatment due to an increase in soluble sugars related to the metabolism (Sadhu et al. 2017). However, the alterations induced by plasma treatment on enzymes may be temporary since the changes in the structure of enzymes partially tend to recover to an untreated state upon storage (Tolouie et al. 2018). To summarize, different parameters affect the enzyme activity after plasma treatment, such as disruption of chemical bonds (such as disulfide and hydrogen bonds), changes in the secondary structure of the enzyme, changes in the PH of the environment, and different chemical modifications, such as ring-opening of amino acids, sulfonation, amidation, hydroxylation, and oxidation (Saremnezhad et al. 2021).

#### Antioxidant activity

Antioxidant activity is not the only indicator of different flavonoids, flavanols, and polyphenols present in food products. The antioxidant effect of various phenolic compounds could be attributed to their redox properties which include potential mechanisms like transition metal-chelating activity, free-radical scavenging activity, and singlet-oxygen quenching capacity (S. Pankaj et al. 2018a, b). Antioxidants are one of the most important bioactive compounds present, which CP treatment can affect. The effect of the CP process on antioxidants depends on many factors, such as the reactivity and type of plasma species and their ability to diffuse into the food matrix. It is documented that antioxidant activity is increased after CP treatment for different foods. After spark discharge plasma treatment, the antioxidant capacity of cloudy apple juice was found to be increased (Saremnezhad et al. 2021). Another study revealed that after the treatment of blueberries with ACP, antioxidant activity was increased (Ji et al. 2020). Also, the antioxidant activity of fresh-cut pitaya fruit is significantly increased after CP treatment (Li et al. 2019). Another study showed that nine out of twelve herbs treated with plasma had higher antioxidant capacity. However, recent studies proved that cold plasma might not affect the antioxidant activity of the food or even reduce it (Pogorzelska-Nowicka et al. 2021). The decrease in antioxidant power could be attributed to the ability of antioxidants to scavenge the plasma-generated free radicals and their decrease in their concentration in the food product. Studies reported the reduction in antioxidant activity of Indica rice paddies after treatment with low-pressure plasma after germination. Also, a loss in antioxidant power of white grape juice after treatment with HVACP due to a reduction in the total phenolic content after treatment (Saremnezhad et al. 2021). Another study reported a reduction in total phenols in orange juice and lamb’s lettuce, while no observed effects in apples. The same study also proved that there was no significant effect on the antioxidant activity in red chicory, kiwifruits, onion powder, sprouts, and radishes (Pankaj et al. 2018a, b).

### Advantages and disadvantages of cold plasma technology

Research has proven that CP treatment offers a wide range of benefits across various food industries, including the ability to combat food-borne microorganisms and neutralize toxins and enzymes (Nwabor et al. 2022). It can be adapted to different food metrics using cost-effective and sustainable technology and it also does not require any chemicals in the process, so there is no heat damage or chemical residue formation (Marshall et al. 2020). Some investigations reported that the CP technique has quick processing time, low-temperature operation, significant antimicrobial activity with little effect on food quality and safety, and excellent energy economy. Furthermore, there were no discernible alterations in the vitamin C content, turbidity, pH, and brix of orange juice treated with plasma (Jiang et al. 2022). A significant study revealed that CP technology is more effective and sufficient than other conventional technologies, such as fumigation, gamma radiation, and steam heating. Additionally, research shows that CP treatment effectively combats pathogens in various spices like paprika powder, black pepper, red pepper flakes, crushed oregano, and onion powder, all without compromising their quality. In summary, cold plasma technology can be effective in multiple stages of the food chain, ensuring both microbial and chemical safety (Ranjan et al. 2023).

Despite the ongoing proof of CP treatment's significant benefits, we still need to discuss the numerous investigations and limitations. For example, it is reported that CP treatment resulted in chemical modifications to dietary ingredients. Many of these dietary ingredients would lose their bioactive properties after treatment. Researchers have also demonstrated the harmful effects of the by-products on human health, including protein modifications, protein structure reduction, sugar oxidation into organic acids, unclear lipid and unsaturated fatty acid peroxidation, and disruption of helical structure into amino acids (Jiang et al. 2022). Another study found that some alterations might result from CP treatment, which leads to unpleasant changes in the physical properties of food and results in economic loss. Researchers also discovered chemical alterations, such as lipid oxidation, in CP-treated mackerel. Mackerel slices showed a reduction in the contents of oleic acid and eicosapentaenoic acid. Also, after CP treatment, thiobarbituric acid reactive substances showed an increase in Asian sea bass slices (Nwabor et al. 2022). CP technology faces some problems, such as a small range of uses, lack of standardisation, low penetration, possible negative effects on food quality, and lack of understanding of how it works (Ranjan et al. 2023). We should evaluate the cytotoxicity of ozone, chlorine, ROS/RNS, and nitrite present in CP-treated food, as well as their oral toxicity. Furthermore, Jiang et al. 2022, reported the mutagenic activity of cold atmospheric plasma-treated protein. CP technology-related ozonolysis processes enhance glycoside bond cleavage, leading to macromolecule depolymerization and functional group oxidation (Varilla et al. 2020). Further research on its mechanism of action needs to be studied to assess the health benefits of the substrates being treated.

### Recent advances of cold plasma treatment

Recent research has explored the combination of cold plasma technology with other methods to enhance the treatment of aflatoxins. This multi-faceted approach aims to improve the efficacy of aflatoxin degradation while maintaining food quality. Combining cold plasma with chemical agents such as ozone has shown promise for increasing the degradation rates of aflatoxins. When ozone and cold plasma are used together, they can make more reactive species that are better at killing aflatoxins. This means that more of them are removed than with either method alone. Jessica Laika and her colleagues, in a recent study, used the system's unique feature, which has the ability to operate at two different power levels, creating two distinct plasma reactive environments: one dominated by ozone (O3) and the other by nitrogen oxides (NOx). The CAP treatments led to significant reductions in the mycotoxins, with the effectiveness varying based on the plasma regime, chemical structure of the molecule, distance from the plasma source, and treatment duration. Specifically, the O3 regime was more effective, achieving a 99% reduction in AFB1 and AFG1, a 60% reduction in AFB2 and AFG2, and a 70% reduction in OTA after 60 min at a 4 cm distance from the plasma source. They selected pistachio kernels, which represent low-moisture foods, to assess the matrix effect. The mycotoxins were less reduced in the food matrix than in their pure forms, with a noteworthy 23% reduction in OTA (Laika et al. 2024). The integration of cold plasma technology with other decontamination methods represents a promising avenue for improving food safety against aflatoxins. Ongoing research aims to optimise these combinations, focussing on scalability and regulatory approval for practical applications in food processing. The goal is to establish effective, safe, and environmentally friendly protocols for aflatoxin mitigation in the food supply chain.

## Conclusion

Aflatoxins remain a formidable challenge in food safety due to their toxicological potency and widespread occurrence in agricultural products. The detailed chemical structure of aflatoxins, particularly AFB1, underscores their carcinogenic potential and adverse health effects associated with human exposure. Current methods of aflatoxin detoxification, including chemical treatments, physical separation, and biological degradation, provide varying degrees of efficacy but often fall short in terms of safety and practicality. The emergence of cold plasma treatment represents a promising advancement in aflatoxin mitigation strategies. Cold plasma technology offers several advantages, including rapid and efficient degradation of aflatoxins, versatility across different food types, and minimal impact on food quality. By harnessing reactive species and physical effects, cold plasma effectively reduces aflatoxin contamination without leaving harmful residues or altering sensory properties. Moving forward, further research is needed to optimize cold plasma parameters, validate its efficacy across diverse food matrices, and ensure regulatory compliance and consumer acceptance. Integration of cold plasma technology into existing food processing systems could significantly enhance food safety measures, particularly in regions prone to aflatoxin contamination. While challenges persist in aflatoxin management, the potential of cold plasma technology offers new hope for improving food safety and public health outcomes worldwide. By leveraging scientific advancements and interdisciplinary collaboration, we can mitigate aflatoxin risks effectively and ensure safer food supplies for the future generations.

## Supplementary Information

Below is the link to the electronic supplementary material.

Supplementary file1 (DOC 198 KB)

## Data Availability

No datasets were generated or analysed during the current study.

## Abbreviations

(CP): Cold plasma
(DBD): Direct barrier discharge
(MW): Micro-wave
(APPJ): Atmospheric pressure plasma jet
(ACP): Atmospheric CP
(HVACP): High voltage atmospheric cold plasma
(APCP): Atmospheric pressure cold plasma
(SEM): Scanning electron microscope
(OH): Hydroxyl radicals
(UV): Ultra Violet
(AFB1): Aflatoxin B1
(CDPJs): Corona discharge plasma jets
(AFB2): Aflatoxin B2
(AFG1): Aflatoxin G1
(RONS): Reactive oxygen and nitrogen species
(ROS): Reactive oxygen species
(ZnONPs): Zinc-oxide nanoparticles
(UFA): Unsaturated fatty acids
(S. Typhimurium): Salmonella typhimurium
(LDH): Lactate dehydrogenase
(O3): Ozone
(Nox): Nitrogen oxides
(IARC): International Agency for Research on Cancer
(AFM1): Aflatoxin M1
(AFG2): Aflatoxin G2
(AFM2): Aflatoxin M2
(AFQ1): Aflatoxin Q1
(HCC): Hepatocellular carcinoma
(TLC): Thin-Layer Chromatography
(HPLC): High-Performance Liquid Chromatography
(PCR): Polymerase Chain Reaction
(ELISA): Enzyme-Linked Immunosorbent Assay
(UPLC–MS/MS): Ultra performance liquid chromatography tandem mass spectrometry
(PKS): Polyketide synthase
(HBx): HBV X protein
(CYP3A4): Cytochrome p450 3A4
(PXR): Pregnane X receptor
(IL11RA-1): IL-11:IL-11 receptor subunit alpha-1
(AF-alb): Albumin adduct levels
(B cells): B lymphocytes
(T cells): T lymphocytes
(ILC2).: Type 2 Innate Lymphoid cells
(PMTDI): Provisional maximum tolerable daily intake
(CKD): Chronic kidney disease
(NAFLD): Non-alcoholic fatty liver disease
(LAB): Lactic acid bacteria
(GRAS): Generally recognized as safe
(NK): Natural killer cells
(HP/HT): High-pressure and high-temperature
(AFs): Aflatoxins
(PDA): Polydopamine
(PVDF): Polyvinylidene fluoride
Ochratoxin A: OTA
