# Supplementary material for: Comprehensive review for aflatoxin detoxification with special attention to cold plasma treatment
Source: Mycotoxin Res. 2025 Feb 1;41(2):277–300. doi: 10.1007/s12550-025-00582-5 (PMC12037664; doi:10.1007/s12550-025-00582-5)
Supplement: Supplementary file 1 — Supplementary file1 (DOC 198 KB) [file 12550_2025_582_MOESM1_ESM.doc]

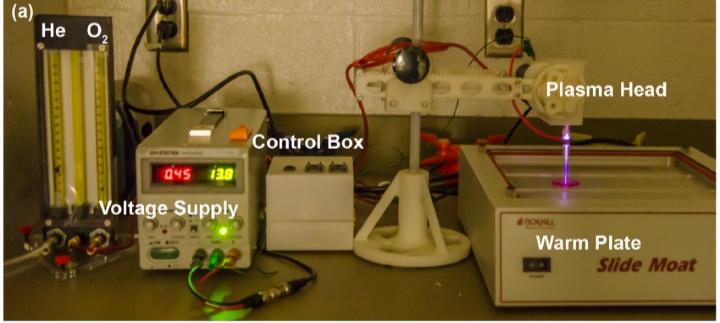


Fig. 1 The cold plasma device setup: voltage supply, control box, plasma head, and flow meter (Cheng et al.2014)


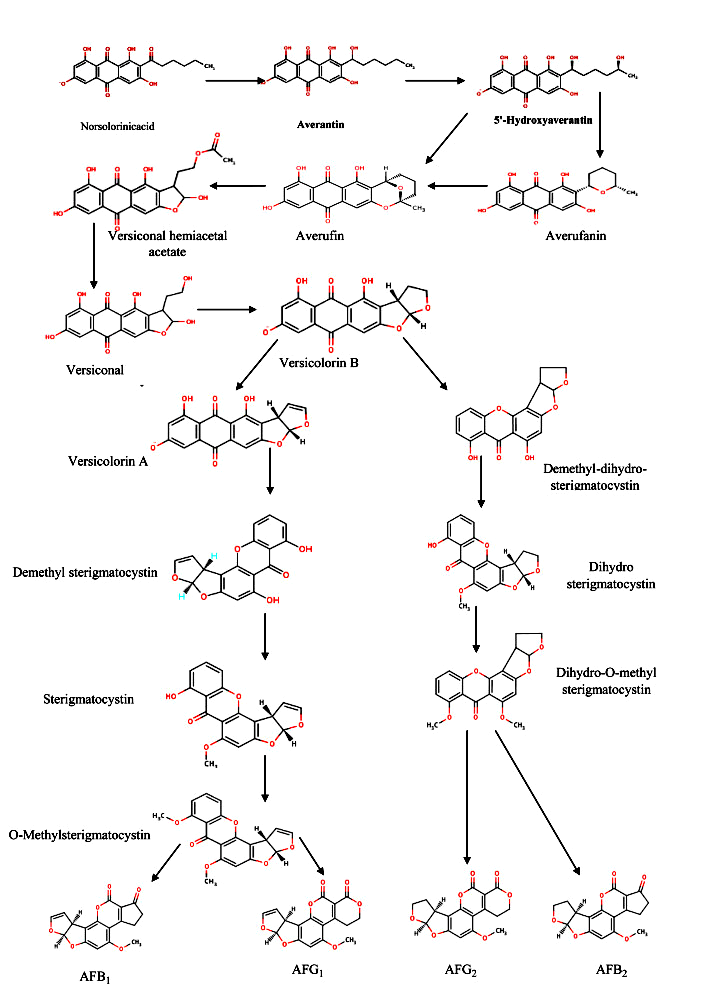


Fig. 2. Biosynthesis of Aflatoxins (Gacem , and El Hadj-Khelil,2016 )

**References:**

Cheng X., J. Sherman, W. Murphy, E. Ratovitski, J. Canady, and M. Keidar, “The Effect of Tuning Cold Plasma Composition on Glioblastoma Cell Viability,” *PLoS ONE*, vol. 9, no. 5, p. e98652, May 2014, doi: 10.1371/journal.pone.0098652.

Gacem MA, A. Ould El Hadj-Khelil, “Toxicology, biosynthesis, bio-control of aflatoxin and new methods of detection,” *Asian Pac. J. Trop. Biomed.*, vol. 6, no. 9, pp. 808–814, Sep. 2016, doi: 10.1016/j.apjtb.2016.07.012.
